# A Porous Aromatic Framework Constructed from Benzene Rings Has a High Adsorption Capacity for Perfluorooctane Sulfonate

**DOI:** 10.1038/srep20311

**Published:** 2016-02-04

**Authors:** Qin Luo, Changwei Zhao, Guixia Liu, Hao Ren

**Affiliations:** 1 State Key Laboratory of Environmental Aquatic Chemistry, Research Center for Eco-Environmental Sciences, Chinese Academy of Sciences, Beijing 100085, P. R. China; 2 Key Laboratory of Applied Chemistry and Nanotechnology at Universities of Jilin Province, Changchun University of Science and Technology, Changchun 130022, P. R. China; 3 State Key Laboratory of Inorganic Synthesis and Preparative Chemistry, Jilin University, Changchun 130012, P. R. China

## Abstract

A low-cost and easily constructed porous aromatic framework (PAF-45) was successfully prepared using the Scholl reaction. PAF-45 was, for the first time, used to remove perfluorooctane sulfonate (PFOS) from aqueous solution. Systematic experiments were performed to determine the adsorption capacity of PAF-45 for PFOS and to characterize the kinetics of the adsorption process. The adsorption of PFOS onto PAF-45 reached equilibrium in 30 min, and the adsorption capacity of PAF-45 for PFOS was excellent (5847 mg g^−1^ at pH 3). The amount of PFOS adsorbed by PAF-45 increased significantly as the cation (Na^+^, Mg^2+^, or Fe^3+^) concentration increased, which probably occurred because the cations enhanced the interactions between the negatively charged PFOS molecules and the positively charged PAF-45 surface. The cations Na^+^, Mg^2+^, and Fe^3+^ were found to form complexes with PFOS anions in solution. Density functional theory was used to identify the interactions between PFOS and Na^+^, Mg^2+^, and Fe^3+^. We expect that materials of the same type as PAF-45 could be useful adsorbents for removing organic pollutants from industrial wastewater and contaminated surface water.

Perfluorooctane sulfonate (PFOS) is a typical perfluorinated compound. It has been produced around the world and used in many industries. PFOS was classified as a persistent organic pollutant in 2009^1^, but its use in a small number of specific industries, such as chrome plating, is still allowed in China. PFOS concentrations of up to 5.7 μg L^−1^ have been found in surface water collected near industrial plants^2^. Therefore, it is very important to find effective techniques for removing PFOS from industrial wastewater and contaminated surface water.

Adsorbents can be used to effectively remove PFOS from wastewater^3^,^4^,^5^, and studies have been conducted in which the abilities of various adsorbents (including carbon materials and alumina^6^,^7^,^8^,^9^,^10^,^11^,^12^,^13^,^14^,^15^,^16^) with different porosities to remove PFOS from wastewater have been determined. There is currently a great deal of interest in porous aromatic frameworks (PAFs). These may be useful for removing organic pollutants from wastewater because of their many potentially useful characteristics, which include a composition consisting only of light elements, high stability—even in moist and aerobic conditions—and large surface area. Another useful characteristic of PAFs is that their structures are easy to control. PAF-type materials have recently been found effective at removing harmful materials such as benzene and toluene from the gas phase^17^,^18^,^19^,^20^,^21^,^22^. However, these materials have not yet been used to treat contaminated water.

In the study described in this paper, we used newly synthesized PAF-45, which was easily constructed using a low-cost catalyst, as a model adsorbent to remove PFOS from aqueous solutions for the first time. Our objective was to characterize the adsorption of PFOS onto PAF-45 using batch adsorption experiments. We systematically investigated the effects of the pH of the solution and the concentrations of different cations in the solution on the adsorption of PFOS by PAF-45. Then, we developed an adsorption scheme that captured the electrostatic interactions (between PFOS molecules and the PAF-45 surface and between adjacent PFOS molecules), non-electrostatic interactions (mainly hydrophobic interactions), and interactions between PFOS molecules and cations at the PAF-45 surface to rationalize the experimental results.

## Results and Discussion

### Characterization of the PAF-45

**Figure 1:**
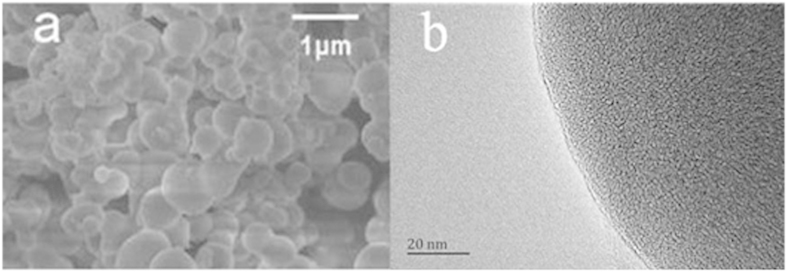
(**a**) Field emission scanning electron microscopy and (**b**) transmission electron microscopy images of PAF-45.

A field emission scanning electron microscopy image of the PAF-45 that was produced is shown in Fig. 1a. The PAF-45 particles were clearly spherical and had diameters of 200–500 nm. The particle and pore morphologies of the PAF-45 were also investigated using transmission electron microscopy, and a representative image is shown in Fig. 1b. The nitrogen adsorption and desorption isotherms for PAF-45 and the distribution of the sizes of its pores are shown in Figure S1 in the Supporting Information [SI]). The PAF-45 had a surface area (Brunauer, Emmett, and Teller) of 873.38 m^2^ g^−1^, a total pore volume of 0.40 mL g^−1^ (for pores with a diameter ≤118.49 nm), a micropore volume of 0.35 mL g^−1^ (for pores with a pore diameter ≤2.07 nm), a point of zero charge of pH 8.80, and a water contact angle of 108°. We previously found that a PFOS molecule is 10.88 Å long^23^. We found that PAF-45 has a narrow range of pore sizes (with two primary maxima centered on 0.56 and 1.3 nm) and that PFOS can be adsorbed onto the surfaces and within the pores of PAF-45.

### The adsorption capacity of PAF-45 for PFOS and the adsorption kinetics

**Figure 2:**
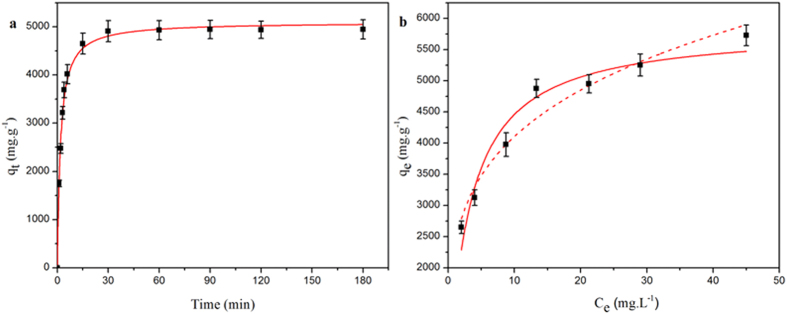
(**a**) Kinetics of the sorption of perfluorooctane sulfonate (PFOS) onto PAF-45 and (**b**) the isotherm for the sorption of PFOS onto PAF-45 with the Langmuir model (dotted line) and Freundlich model (solid line) fitted to the data. Adsorption conditions: the pH was 3, the adsorption temperature was 25 °C, and the initial concentration of PFOS was 100 mg L^−1^. The error bars show the standard deviations obtained by performing triplicate tests.

Many adsorbents have been used to remove PFOS from water, and the adsorption capacities that have been found for a range of adsorbents are summarized in Table 1. In the study presented in this paper, we demonstrated for the first time that PAF-45 has a remarkable capacity for adsorbing PFOS from water. As shown in Fig. 2, the adsorption of PFOS onto PAF-45 almost reached equilibrium within 30 min. The pseudo-second-order rate equation constant, k_2_, for the adsorption of PFOS onto PAF-45, which was determined using a nonlinear model, is shown in Table 1. The pseudo-second-order model gave an adsorption capacity, q_e_, of 5096.17 mg g^−1^, an initial adsorption rate, ν_0_, of 2842.75 mg g^−1^ min^−1^, and a final pH of 3.08, and the coefficient of determination, r^2^, was 0.995. The data were clearly described better by the Freundlich model (which gave a *K* value of 2000 mg^(1−1/n)^ L^1/n^ g^−1^, an *n*^*−1*^ value of 0.2, and an r^2^ value of 0.953) than by the Langmuir model (which gave a q_m_ value of 5847.39 mg g^−1^, a *b* value of 0.32 L mg^−1^, and an r^2^ value of 0.942) over all the PFOS concentration range that was tested and especially at high concentrations. The adsorption capacity of PAF-45 for PFOS is, to the best of our knowledge, much higher than the adsorption capacity of any adsorbent that had previously been tested. We also tested the adsorption capacity of PAF-45 for PFOS in neutral condition, the results indicated that the high adsorption capacity of PAF-45 was obtained at pH 7 (see Figure S6 in SI). The high adsorption capacity of PAF-45 can be ascribed to (i) its hydrophobic aromatic rings (see Figure S2 in SI), which match the hydrophobic C–F chains in PFOS and (ii) its positive charge at pH 3 (see Figure S3 in SI). These characteristics mean that PAF-45 can interact with the SO_3_^−^ group in PFOS through electrostatic attraction^24^,^25^. It is possible for hemi-micelles and even micelles to form within the pores of PAF-45 once many PFOS molecules have been adsorbed because the PFOS concentration is likely to be much higher in the pores than in the solution. This causes the adsorption capacity of PAF-45 to be higher than the adsorption capacities of other adsorbents.

### Reusability of PAF-45

**Figure 3:**
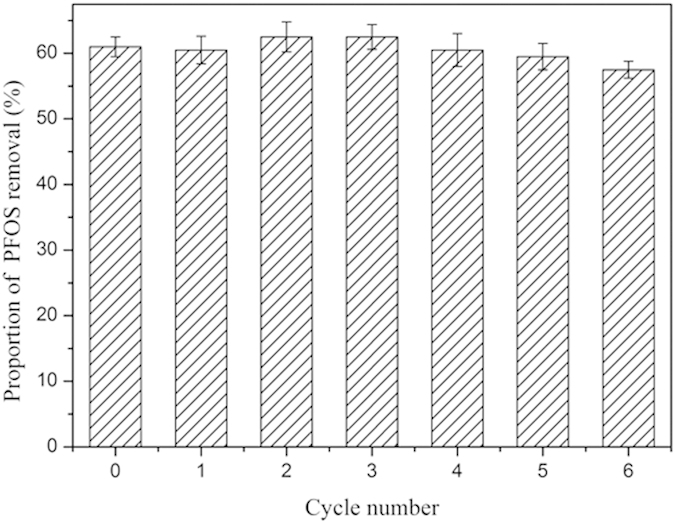
The proportion of perfluorooctane sulfonate (PFOS) removed by PAF-45 in six adsorption–regeneration cycles. The error bars show the standard deviations determined by performing triplicate tests (test conditions: the pH was 3, the adsorption temperature was 25 °C, and the initial concentration of PFOS was 100 mg L^−1^).

PAF-45 was used repeatedly to adsorb PFOS from a solution; it was regenerated between adsorption tests by treating it with a mixture of NaOH and acetone and then, rinsing it with deionized water. The rates at which PFOS was removed from solution by PAF-45 in six consecutive adsorption–regeneration cycles are shown in Fig. 3. We found that PAF-45 could be used in at least six cycles without losing any of its capacity to adsorb PFOS, which indicates that the adsorption properties of PAF-45 were completely refreshed by the regeneration process.

### Effects of pH and the ionic strength of the solution on PFOS adsorption by PAF-45

**Figure 4:**
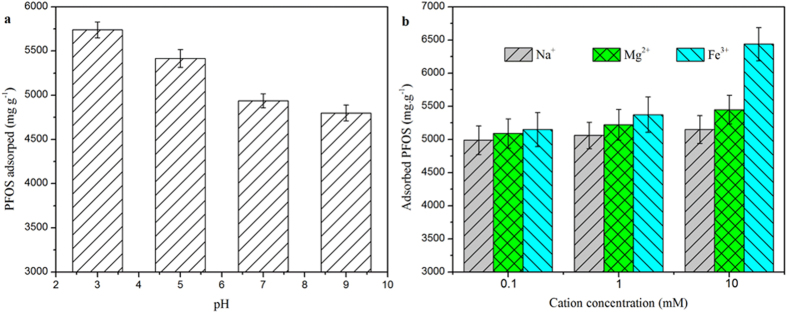
Effects of (**a**) the pH and (**b**) the concentrations of different cations (at pH 3) on the amount of perfluorooctane sulfonate (PFOS) adsorbed by PAF-45 (test conditions: 0.5 mg PAF-45 with an initial PFOS concentration of 100 mg L^−1^ and 48 h of adsorption time). The error bars show the standard deviations determined by performing triplicate tests.

The adsorption of PFOS onto PAF-45 was strongly affected by both the pH and the ionic strength of the solution^26^,^27^,^28^,^29^,^30^,^31^,^32^,^33^,^34^,^35^,^36^,^37^. As shown in Fig. 4a, the adsorption capacity of the PAF-45 increased as the pH decreased, changing from 4814 mg g^−1^ at pH 9 to 5721 mg g^−1^ at pH 3. The PAF-45 was found to be positively charged at acidic pH values, and its point of zero charge was pH 8.8 (Figure S3 in SI). The increase in the capacity of PAF-45 to adsorb PFOS as the pH decreased was probably related to the increase in the strength of the electrostatic attraction as the pH decreased^10^,^38^,^39^,^40^,^41^,^42^. Non-electrostatic interactions (mainly hydrophobic interactions) tend to contribute more than electrostatic interactions to the amount of a species adsorbed, but adsorption through non-electrostatic interactions tends to be little affected by changes in the pH of a solution.

**Figure 5:**
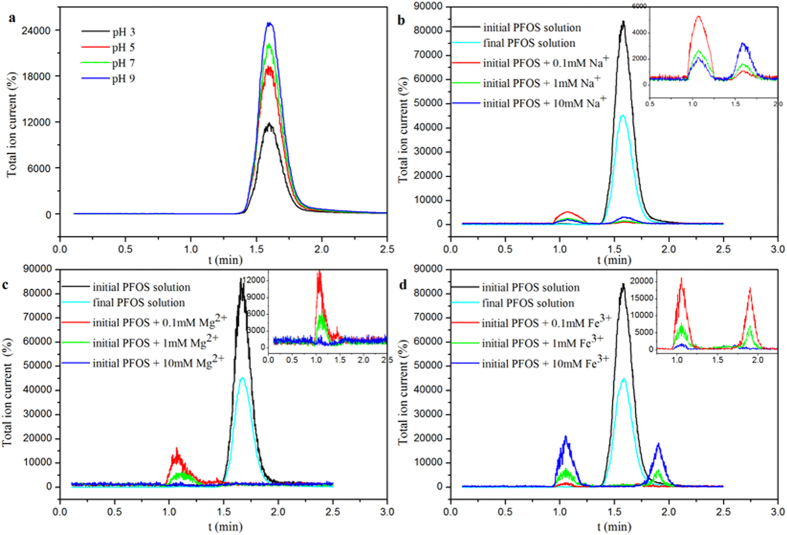
Effects of (**a**) the pH, (**b**) the Na^+^ concentration at pH 3, (**c**) the Mg^2+^ concentration at pH 3, and (**d**) the Fe^3+^ concentration at pH 3 on the liquid chromatography-tandem quadrupole mass spectrometry response to perfluorooctane sulfonate (PFOS). (Test conditions: 0.5 mg PAF-45 with an initial PFOS concentration of 1 mg L^−1^ and 48 h of adsorption time). The insets show magnified versions of the mass spectrometry results after the addition of cations in different concentrations.

The ionic strength of the solution and the type of cation present within it had critical effects on the adsorption of PFOS onto PAF-45. The effects of the presence of Na^+^, Mg^2+^, or Fe^3+^ at different concentrations on the adsorption of PFOS by PAF-45 were determined at pH 3, and the results are shown in Fig. 4b. More PFOS was adsorbed and less time was taken reach equilibrium when any of the cations tested were present than when none of the cations tested were present. Surprisingly, the adsorption capacity of PAF-45 for PFOS was higher (reaching 6439 mg g^−1^) in the presence of Fe^3+^ than in the presence of Na^+^ or Mg^2+^. Such a high adsorption capacity for PFOS had not previously been reported for any other adsorbent. Ultrahigh performance liquid chromatography-tandem quadrupole mass spectrometry (UPLC-MS/MS) chromatograms of PAF-45 with PFOS adsorbed to it in the presence of Na^+^, Mg^2+^, and Fe^3+^ are shown in Fig. 5, and the Fourier transform infrared spectra and X-ray diffraction patterns of PAF-45 under the same conditions are shown Figures S4 and S5 in SI, respectively. As we can see from Figures S7 and S8 in SI, between PFOS and cations (Na^+^, Mg^2+^, or Fe^3+^), PFOS is the priority to interact PAF-45 whether stimulated PFOS or real PFOS contaminants^43^.

Several factors contribute significantly to the effects the presence of the cations tested have on the adsorption capacity of PAF-45. First, there are electrostatic interactions between the cations, the PFOS on the surface, and the PAF-45 surface as well as between neighboring PFOS molecules. The net effect of the chemistry of the solution on a positively charged adsorbent such as PAF-45 is governed by competition between PFOS–surface attraction and PFOS–PFOS repulsion. Increasing the ionic strength of the solution increases the density of positive charges on the PAF-45 surface, enhancing the electrostatic attraction between PFOS and the surface and weakening the electrostatic repulsion between PFOS molecules. Second, the presence of cations has little effect on the non-electrostatic interactions, such as hydrophobic interactions^44^,^45^,^46^, between PFOS and PAF-45 because of the strongly hydrophobic nature of the PFOS perfluoroalkyl chain and some of the PAF-45 moieties. Hydrophobic interactions may occur between the hydrophobic chain of a PFOS molecule and a hydrophobic moiety on the PAF-45 surface or between the hydrophobic chains of different PFOS molecules. Such non-electrostatic interactions are very weakly dependent on or virtually independent of the solution chemistry, and they are the dominant contributors to the removal of PFOS from solution by PAF-type adsorbents, which distinguishes this type of adsorbent from others^47^. Finally, the chemical complexation initiated by cations in the solution affects the ability of PAF-45 to adsorb PFOS.

**Figure 6:**
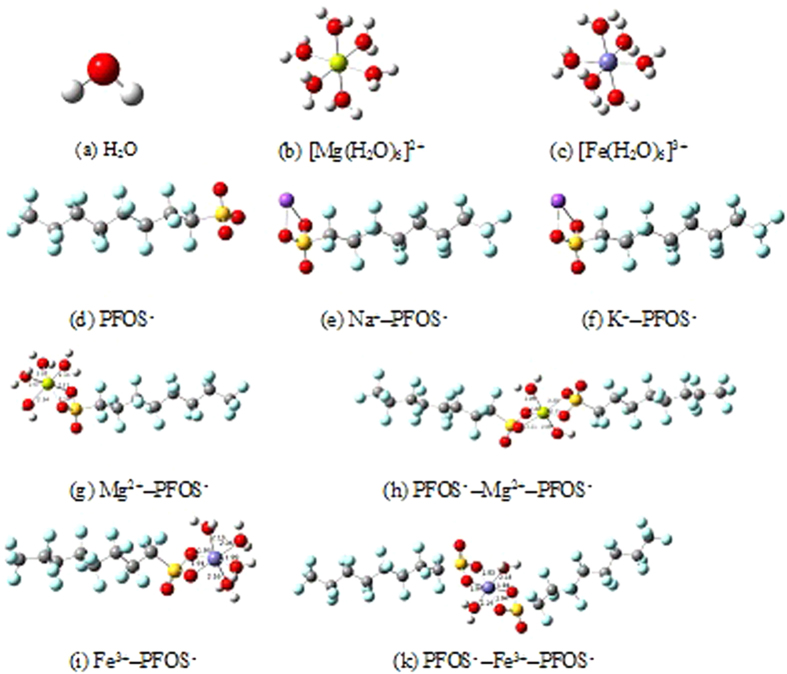
Structures of (**a**) H_2_O, (**b**) Mg(H_2_O)_6_^2+^, and (**c**) Fe(H_2_O)_6_^3+^ and (**d**) the HOMO orbitals of perfluorooctane sulfonate (PFOS), (**e**) the geometry of an Na^+^ ion bound to one PFOS molecule, (**f**) the geometry of a K^+^ ion bound to one PFOS molecule, (**g**) the geometry of an Mg^2+^ ion bound to one PFOS molecule, (**h**) the geometry of an Mg^2+^ ion bound to two PFOS molecules, (**i**) the geometry of an Fe^3+^ ion bound to one PFOS molecule, and (**k**) the geometry of an Fe^3+^ ion bound to two PFOS molecules. The isosurface plots of the HOMO orbitals were generated using an isodensity value of 0.02 a.u.

The zeta potential became positive (see Figure S3 in SI) when a cation was added, indicating that cations were adsorbed onto the PAF-45 surface. We have previously found that bridging can occur between Mg^2+^ ions and the sulfonate functional groups in PFOS molecules, and we have quantified the calcium-bridging mechanism using Density functional theory calculations^23^. The presence of Fe^3+^ was found to decrease the UPLC-MS/MS response to PFOS, which indicates that PFOS may have formed a complex with the Fe^3+^ 48. The UPLC-MS/MS total ion current of a sample with a known PFOS concentration was significantly lower when one of the test cations was present than when the test cations were absent, and new peaks were formed. The UPLC-MS/MS results shown in Fig. 5 show that PFOS could be adsorbed while coordinated with Na^+^ (see Fig. 5b), Mg^2+^ (see Fig. 5c), or Fe^3+^ (see Fig. 5d). This indicates that the cations may have formed complexes with the PFOS, decreasing the concentration of non-complexed PFOS (measured by UPLC-MS/MS). The geometries of the structures formed when PFOS interacts with the different cations, from Density functional theory calculations, are shown in Fig. 6, and the thermodynamic parameters and the dipole moment parameters are listed in Table 2.

### Thermodynamics of the adsorption processes

The structure of H_2_O is shown in Fig. 6a, and the structures of [Mg(H_2_O)_6_]^2+^ and [Fe(H_2_O)_6_]^3+^, which are the stable species of Mg^2+^ and Fe^3+^ in aqueous solution, are shown in Fig. 6b,c, respectively. Na^+^ and K^+^ are strongly ionized in water; therefore, they are present as free ions. A frontier orbital analysis suggested that the HOMO of the anionic PFOS surfactant is primarily localized on the sulfonate head group (Fig. 6d), which indicates that the –SO_3_ group is the active site that binds to the positively charged Na^+^, Mg^2+^, and Fe^3+^ ions. Mg^2+^ and Fe^3+^ ions may bind to one or two PFOS anions to create bridged structures. The reactions between PFOS and K^+^, Na^+^, Mg^2+^, and Fe^3+^ are shown, with the changes in their Gibbs free energies (ΔG^θ^), in reactions 1–6.


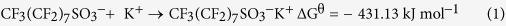



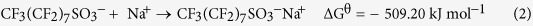



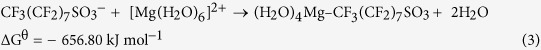



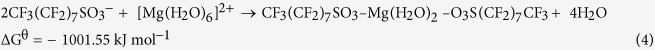



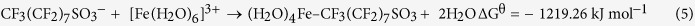



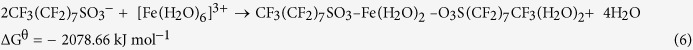


The Gibbs free energy changes for all the reactions are negative, which shows that all these reactions occur spontaneously. The commercially available form of PFOS is PFOS^−^K^+^. The Gibbs free energy change is lower for reaction 1 than for reaction 2, which indicates that the form CF_3_(CF_2_)_7_SO_3_Na is energetically more favorable than the form CF_3_(CF_2_)_7_SO_3_K (Fig. 6e). Density functional theory calculations showed that the configurations CF_3_(CF_2_)_7_SO_3_Mg(H_2_O)_4_ (Fig. 6g) and CF_3_(CF_2_)_7_SO_3_MgO_3_S(CF_2_)_7_CF_3_(H_2_O)_2_ (Fig. 6h) form when Mg^2+^ binds with one or two PFOS molecules, respectively. We found that PFOS bound to Mg^2+^ is more polar than unbound PFOS (*μ* = 22.9 for PFOS, but *μ* = 31.2 for CF_3_(CF_2_)_7_SO_3_Mg(H_2_O)_4_), which means that interactions between Mg^2+^-bound PFOS and the PAF-45 surface are more favorable than interactions between unbound PFOS and the PAF-45 surface. This is in agreement with the higher adsorption efficiency that was found when Mg^2+^ was present. The Gibbs free energy changes for reactions 3 and 4 were found to be −656.80 and −1001.55 kJ mol^−1^, respectively (also see Table 2), which suggests that the formation of CF_3_(CF_2_)_7_SO_3_MgO_3_S(CF_2_)_7_CF_3_(H_2_O)_2_ is favorable. The configurations of Fe^3+^ bound to one and two PFOS molecules are shown in Fig. 6I,k, respectively. Three PFOS molecules cannot bind to Fe^3+^ due to steric hindrance. The Gibbs free energy changes for reactions 5 and 6 were found to be −1219.26 and −2078.66 kJ mol^−1^, respectively, which suggests that an Fe^3+^ ion easily forms a bridge between two PFOS anions by generating an attractive force that is effective at the length of an Fe–O(S) bond. This attraction is sufficiently strong to cause the PFOS molecules to be effectively linked together through a bridge-like PFOS–Fe–PFOS configuration. Our calculations show that Fe^3+^ is more able than Na^+^ or Mg^2+^ to form a bridge between PFOS molecules. This is consistent with our finding that the PFOS adsorption equilibrium was reached more quickly in the presence of Fe^3+^ than in the presence of Na^+^ or Mg^2+^.

## Conclusions

The PAF-45 produced was found to be very stable in moist aerobic conditions and to contain uniform pores. We found that PFOS could diffuse into and adsorb onto PAF-45. The adsorption of PFOS onto PAF-45 reached equilibrium within 30 min, and the adsorption capacity of PAF-45 for PFOS at pH 3.0 was 5847 mg g^−1^. We attributed the high adsorption capacity of PAF-45 for PFOS to PAF-45’s positively charged surface and extremely hydrophobicity. The pH had a significant effect on the amount of PFOS adsorbed, and the composition of the solution affected the amount of PFOS adsorbed and the kinetics of the adsorption process. We found that certain cations can form bridges between PFOS molecules. Our results show that PAFs have a remarkable amount of potential for removing pollutants from contaminated water.

## Methods

### Chemicals and general information

Potassium PFOS (99% pure) was purchased from AccuStandard. HPLC-grade methanol and acetonitrile were purchased from Fisher Scientific. Sodium chloride, magnesium chloride, iron chloride, sodium hydroxide, and hydrochloric acid were purchased from Sinopharm Chemical Reagent Company. Biphenyl (99% pure) was provided by Aladdin. Other materials were purchased from commercial suppliers and used without further purification unless otherwise noted. The ultrapure water used was produced using a Milli-Q integral water purification system.

The morphology of the PAF-45 was examined using a Hitachi S-3000N field emission scanning electron microscope and a JEOL JEM-2100F transmission electron microscope. The crystallinity and regularity of the PAF-45 were determined by X-ray diffractometry using an AXS instrument. The surface area of the PAF-45 was determined using the Brunauer, Emmett, and Teller method. The functional groups were identified using Fourier transform infrared spectroscopy. The zeta potential was determined at different pH values. The pH was measured using a pH meter. The contact angle of the PAF-45 was determined using a DataPhysics DCAT21 dynamic contact angle measuring instrument.

### Synthesis of PAF-45 nanoparticles

PAF-45 was prepared as described in a previous publication^17^,^49^,^50^. Anhydrous aluminum chloride was added to a round-bottomed flask, and then, the flask was attached to a vacuum to remove the gases that were present. The flask was then filled with N_2_, and the gas was evacuated three times. Dry CHCl_3_ (60 mL) was then injected using a syringe, and the mixture was kept at 60 °C for 3 h. Biphenyl in CHCl_3_ (40 mL) was added to the flask, and the mixture was kept at 60 °C for 24 h while being stirred. The contents were cooled to room temperature and filtered to separate the crude product, which was washed with hydrochloric acid, then methanol, and then acetone to remove unreacted monomers and catalyst residues. The product was further purified using Soxhlet extraction with ethanol, tetrahydrofuran, and trichloromethane, successively, for 48 h each.

### Sorption experiments

The sorption experiments were conducted in polypropylene centrifuge tubes (50 mL), each of which contained PAF-45 (0.5 ± 0.02 mg) and a PFOS solution (40 mL) of the required concentration. The tubes were shaken at 180 rpm and kept at 25 °C for 48 h. The pH of the solution in each tube was adjusted using 0.1 M HCl and 0.1 M NaOH, and the ionic strength was controlled by adding 1 M NaCl_(aq)_, MgCl_2(aq)_, or FeCl_3(aq)_. The initial PFOS concentration used in the adsorption kinetics experiments was 100 mg L^−1^. The adsorption isotherm experiments were conducted using PFOS concentrations of 50–200 mg L^−1^, and the solutions were kept at pH 3 by making regular adjustments during the sorption experiments. All the tests were performed in triplicate.

### Regeneration experiments

An aliquot of PAF-45 (0.5 ± 0.02 mg) was placed in a PFOS solution (40 mL, 100 mg L^−1^) at pH 3 for 48 h, and then, the PAF-45 with adsorbed PFOS was separated from the mixture. The PAF-45 was then regenerated by mixing it with a mixture of NaOH_(aq)_ (0.5 M) and acetone (90:10 v/v, 40 mL) in a thermostatic shaker at 40 °C for 24 h and then, rinsing it with deionized water until the washings reached a neutral pH. The regenerated PAF-45 was freeze dried (although it may be possible to use the adsorbent wet in actual applications) and reused in the next sorption cycle of the sorption–regeneration experiment.

### PFOS determination

After each sorption experiment, the mixture was passed through a 0.22 μm nylon membrane filter and diluted until its PFOS concentration was 1000 μg L^−1^. The PFOS concentration of the solution was determined using an UPLC instrument coupled to a Quattro Premier XE MS/MS system. The amount adsorbed was defined as the difference between the PFOS concentrations of the solution before and after sorption, and the removal rate (the percentage of the starting amount removed) was calculated.

## Additional Information

**How to cite this article**: Luo, Q. *et al*. A Porous Aromatic Framework Constructed from Benzene Rings Has a High Adsorption Capacity for Perfluorooctane Sulfonate. *Sci. Rep*. **6**, 20311; doi: 10.1038/srep20311 (2016).

## Supplementary Material

Supplementary Information

## Figures and Tables

**Table 1:** Adsorption capacities of different adsorbents for perfluorooctane sulfonate and kinetic parameters for the adsorption of PFOS by each adsorbent.

Adsorbent	C_0_^a^ (mg L^−1^)	pH	T_equi_^b^ (h)	k_2_^c^ (g mg^−1^ h^−1^)	q_m_^d^ (mg g^−1^)/(m^2^ g^−1^)	S^e^(m^2^/g)	Refs
Granules of activated carbon	15–250	4.4–7.2	48–168	1.4 × 10^−4^	0.22–0.29	780	3,4,6
Zeolites	15–300	6.8–7.2	3–5	1.15–3.69	0.01–0.16	800	3,13
Hydrotalcite	1–1000	-	~1	31.77	~4.99	200	31
Maize straw-origin ash	1–500	7	48	2.2 × 10^−3^	21.17^e^	38.3	14
Crosslinked chitosan beads	46–371	3	~100	-	194.68	14.1	10
PAF-45	50–200	3	0.5	1.09 × 10^−4^	6.70	873.4	this study

^a^Initial concentration;

^b^Equilibrium time;

^c^Pseudosecond-order rate constant;

^d^Adsorption capacity in terms of the specific surface area;

^e^Surface area.

**Table 2:** Thermodynamic parameters and dipole moments (μ) for H_2_O, K^+^, Na^+^, Mg^2+^, Fe^3+^ and for the structures formed when PFOS interacts with K^+^, Na^+^, Mg^2+^, and Fe^3+^, calculated using density functional theory.

Structures	Dipole moment *μ*(Debye)	ΔG (kJ mol^−1^)	S (kJ mol^−1^ K^−1^)
H_2_O	2.09	−200.60	0.188849
K^+^	0	−1574.62	0.155018
Na^+^	0	−425.58	0.148411
[Mg(H_2_O)_6_]^2+^	0	−1727.97	0.507783
[Fe(H_2_O)_6_]^3+^	0	−4518.60	0.490683
CF_3_(CF_2_)_7_SO_3_^−^	22.9	−6894.32	0.771433
CF_3_(CF_2_)_7_SO_3_Na	8.4	−7320.41	0.817702
CF_3_(CF_2_)_7_SO_3_K	10.5	−8469.37	0.824313
CF_3_(CF_2_)_7_SO_3_Mg(H_2_O)_4_	31.2	−8221.74	1.014294
CF_3_(CF_2_)_7_SO_3_Mg(H_2_O)_2_O_3_S(CF_2_)_7_CF_3_	2.75	−14715.20	1.500454
CF_3_(CF_2_)_7_SO_3_Fe(H_2_O)_4_	42.5	−11012.64	1.024105
CF_3_(CF_2_)_7_SO_3_Fe(H_2_O)_2_O_3_S(CF_2_)_7_CF_3_	3.90	−17506.91	1.578138
