# Supplementary material for: A Porous Aromatic Framework Constructed from Benzene Rings Has a High Adsorption Capacity for Perfluorooctane Sulfonate
Source: Sci Rep. 2016 Feb 4;6:20311. doi: 10.1038/srep20311 (PMC4740861; doi:10.1038/srep20311)
Supplement: Supplementary Information [file srep20311-s1.doc]

**Supplementary information**

A Porous Aromatic Framework Constructed from Benzene Rings Has a High Adsorption Capacity for Perfluorooctane Sulfonate

Qin Luo^1,2^, Changwei Zhao^1,*^, Guixia Liu^2^, and Hao Ren^3^

^1^State Key Laboratory of Environmental Aquatic Chemistry, Research Center for Eco-Environmental Sciences, Chinese Academy of Sciences, Beijing 100085, P. R. China, ^2^Key Laboratory of Applied Chemistry and Nanotechnology at Universities of Jilin Province, Changchun University of Science and Technology, Changchun 130022, P. R. China, ^3^State Key Laboratory of Inorganic Synthesis and Preparative Chemistry, Jilin University, Changchun 130012, P. R. China.


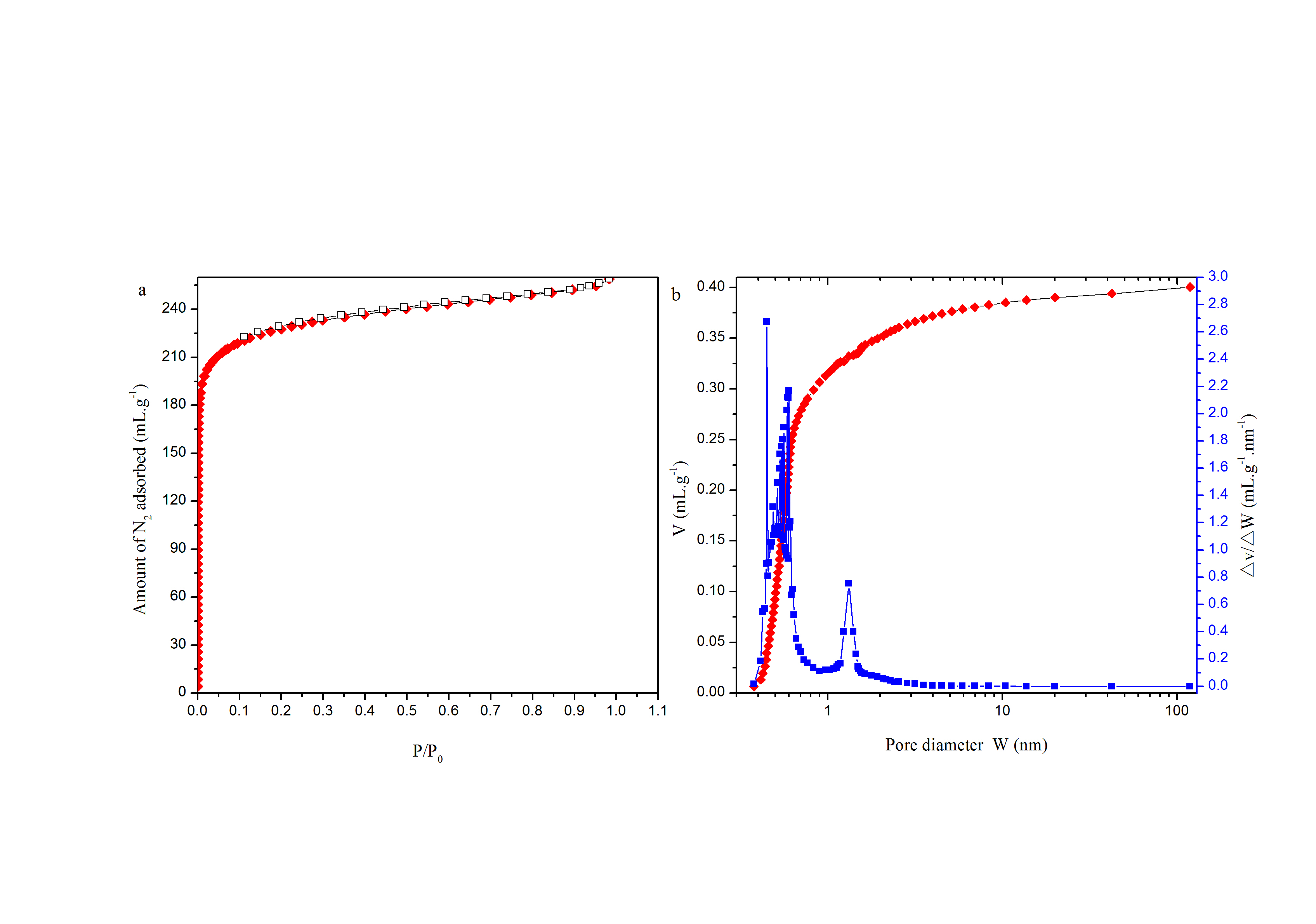


Figure S1 **|** (a) N2 adsorption isotherms for PAF-45 (solid diamonds for adsorption and open squares for desorption) and (b) the distribution of the diameters of the pores in PAF-45.


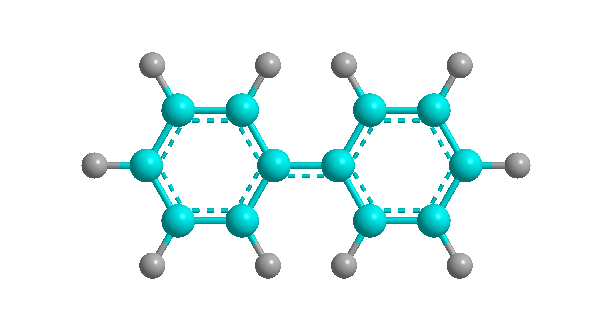

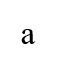

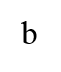

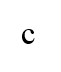


Figure S2 **|** (a) Monomer used to construct the PAF-45, (b) Scholl reaction, and (c) the uncertain structures formed through multiple substitutions in the PAF-45(blue represents meta-substitution, while red and green stand for para-substitution and ortho-substitution, respectively)


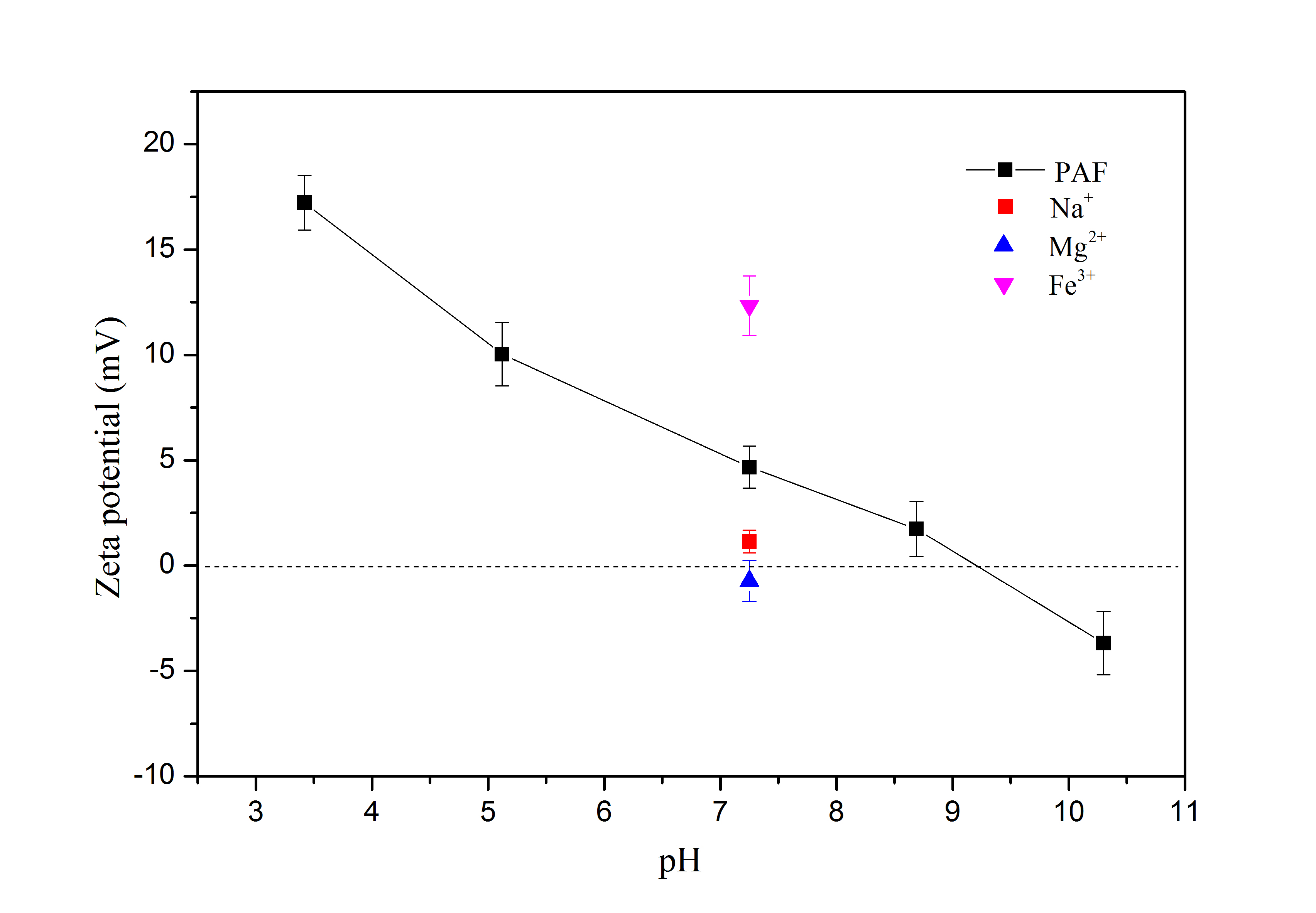


Figure S3 **|** Isoelectric point of the virgin PAF-45 nanoparticle and after addition of different cations at different pH values. The error bars show the standard deviations determined by performing triplicate tests (test condition: 0.5 mg PAF-45 with 10 mM cation initial concentration).

As can be seen from Figure S2, the PAF-45 was positively charged at neutral pH values, and the point of zero charge was pH 8.8. The zeta potential became positive when cations were added at neutral pH, indicating that the cations must interact with the PAF-45.


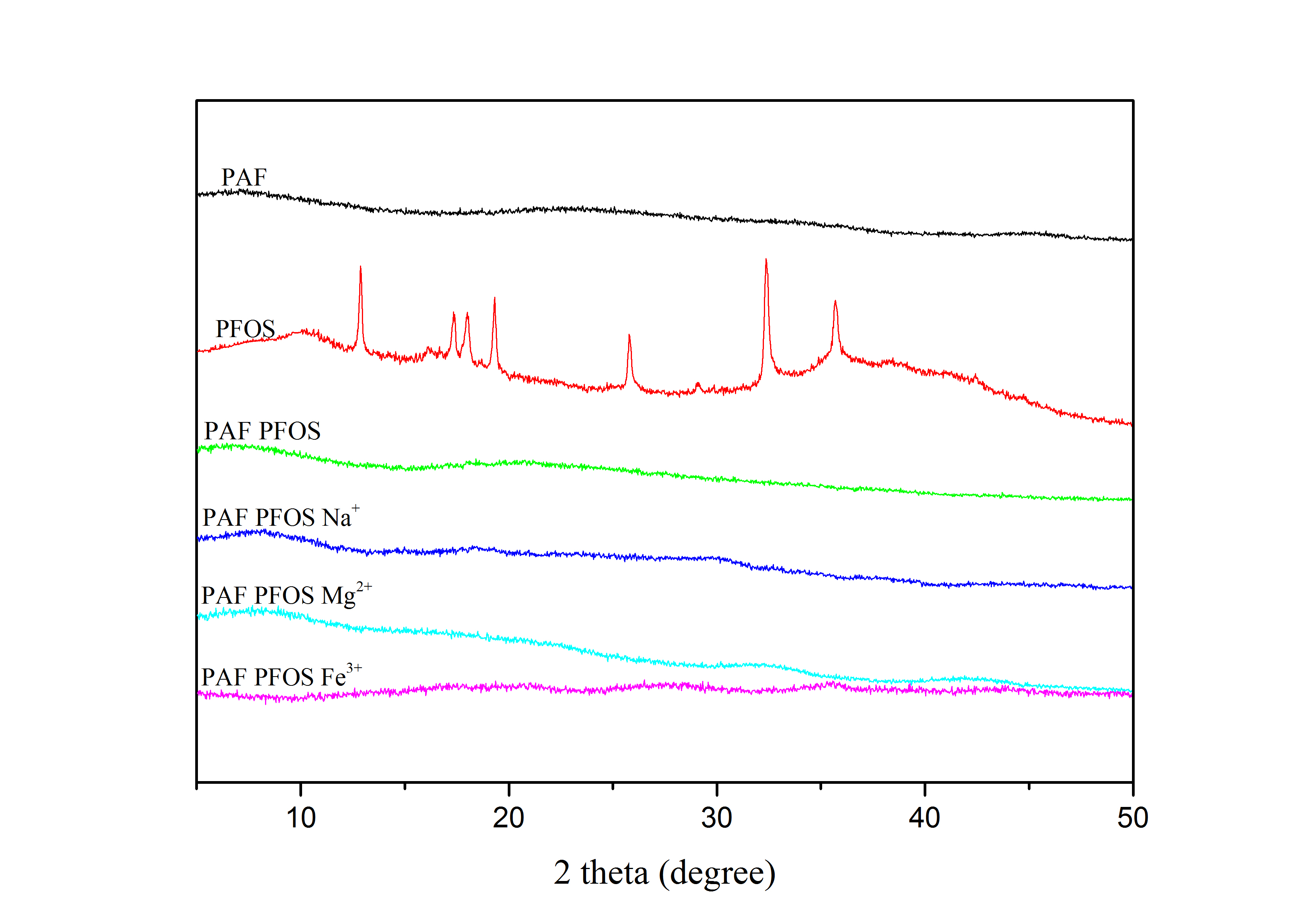


Figure S4 **|** X-ray diffraction patterns for the PAF-45, perfluorooctane sulfonate (PFOS), PAF-45–PFOS, PAF-45–PFOS–Na^+^, PAF-45–PFOS–Mg^2+^, PAF-45–PFOS–Fe^3+^ (test condition: 0.5 mg PAF-45 with 10 mM cation initial concentration and 1 mg.L^-1^ PFOS initial concentration).

The crystallinity of the PAF-45 with and without PFOS adsorbed to it was investigated by XRD, and the results are shown in Figure S3. The XRD patterns showed that the as- synthesized PAF-45 was amorphous and that it remained amorphous when PFOS and the cations tested (Na^+^, Mg^2+^ and Fe^3+^) were added. However, the peak shape changed when PFOS and the cations tested (Na^+^, Mg^2+^ and Fe^3+^) were added, indicating that there must have been interactions between the PAF-45 and the PFOS, Na^+^, Mg^2+^ and Fe^3+^. Therefore, we studied these interactions further.


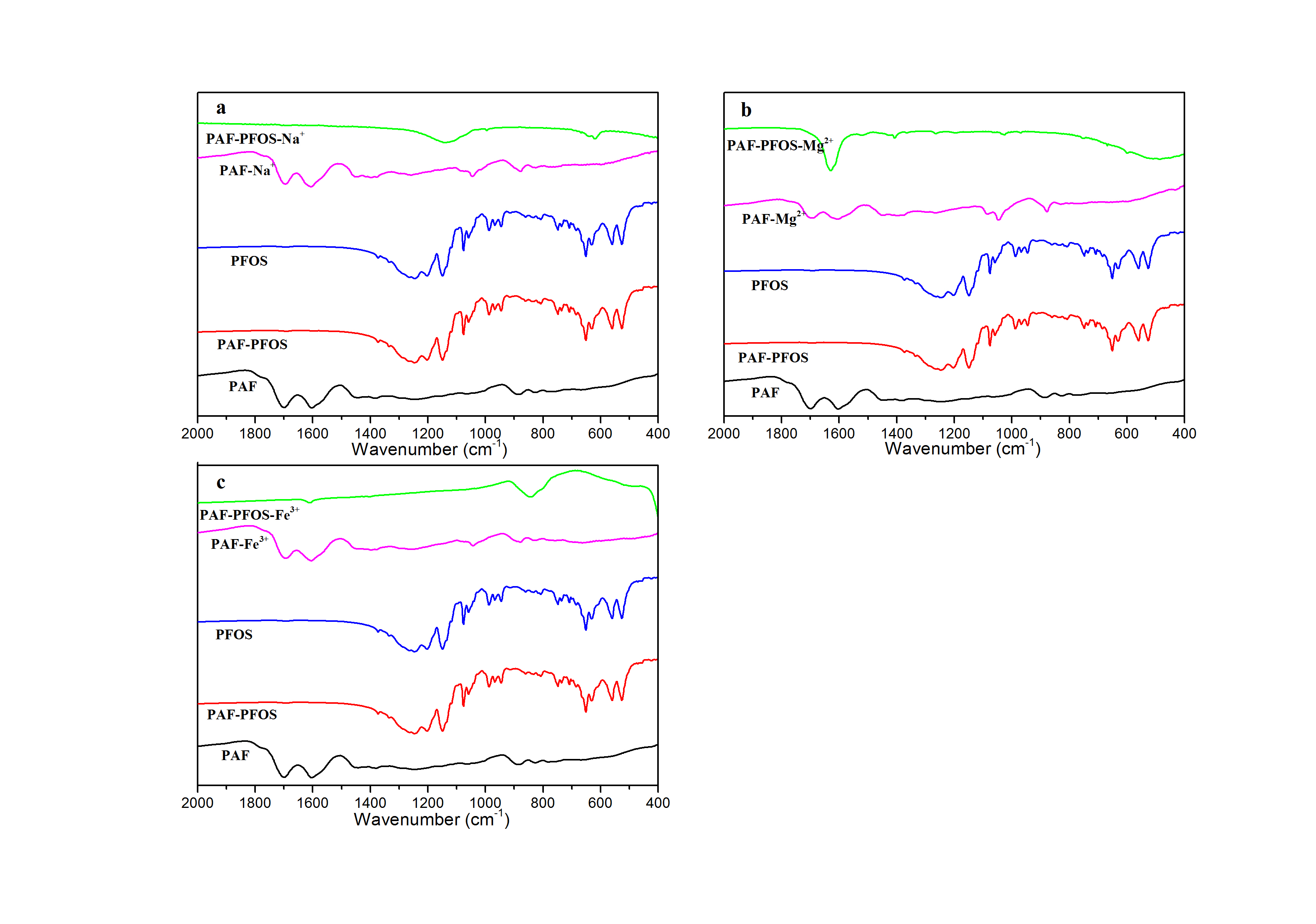


Figure S5 | FT-IR spectra from 2000 cm^-1^ to 400 cm^-1^ of (a) PAF-45–perfluorooctane sulfonate (PFOS)–Na^+^, (b) PAF-45–PFOS–Mg^2+^, and (c) PAF-45–PFOS–Fe^3+^, used to study the interactions between PAF-45, PFOS and the cations (test condition: 0.5 mg PAF-45 with 10 mM cation initial concentration and 1 mg.L^-1^ PFOS initial concentration).

The FT-IR spectra shown in Figure S4 was acquired to allow the interactions between the PAF-45, PFOS and the cations to be investigated. As can be seen from Figure S4 a, b, and c, the spectrum was the same for the PAF-45 that had adsorbed PFOS and for PFOS alone, indicating that the PFOS completely covered the PAF-45. The absorption peaks in the 1650–1400 cm^-1^ region and 910–860 cm^-1^ regions were assigned to vibrations of the C=C bonds and isolated hydrogen atoms in the PAF-45 benzene rings, respectively. The C=C absorption band was not found in the PAF-45–PFOS–cation spectra, and this was attributed to the formation of PAF-45–PFOS–cation networks causing the stretching and deformation vibrations of the molecules to be restricted. The PAF-45 peak at 887 cm^-1^ (for C–H deformation vibrations of isolated hydrogen atoms) was clearly red-shifted in the PAF-45–cation spectra, and this was attributed to the formation of p-π conjugates between the benzene rings and the cations.


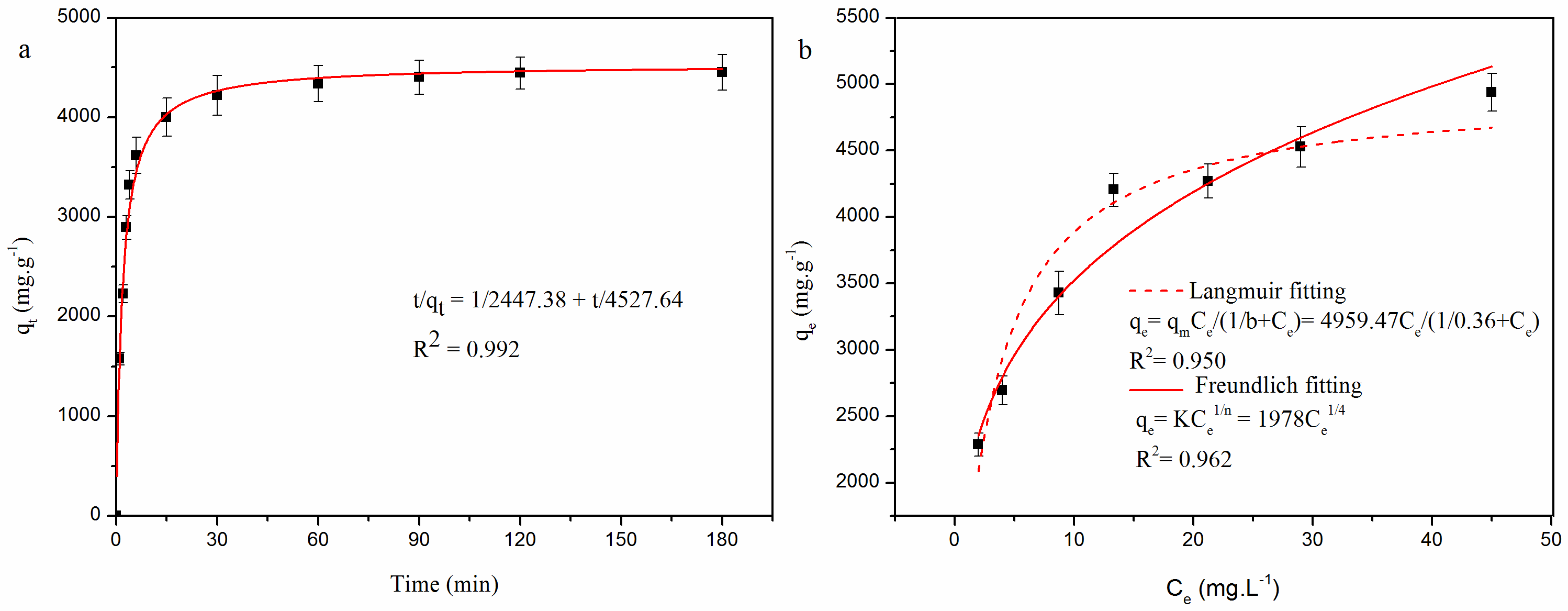


Figure S6 | Kinetics of the sorption of perfluorooctane sulfonate (PFOS) onto PAF-45 and (b) the isotherm for the sorption of PFOS onto PAF-45 with the Langmuir model (dotted line) and Freundlich model (solid line) fitted to the data. Adsorption conditions: the pH was 7, the adsorption temperature was 25℃, and the initial concentration of PFOS was 100 mg L^−1^. The error bars show the standard deviations obtained by performing triplicate tests.


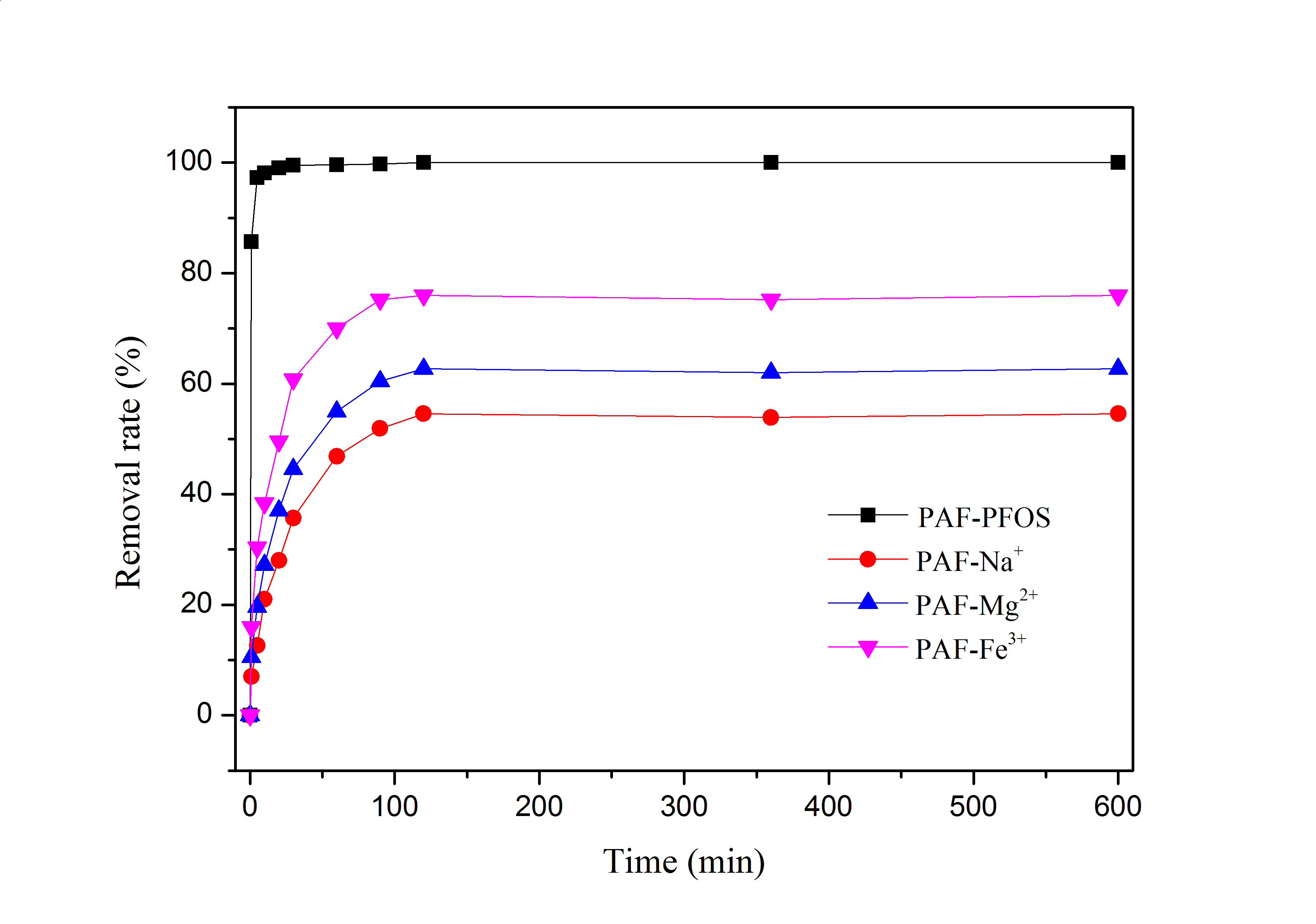


Figure S7 | Reaction kinetics of PAF-PFOS (black), PAF-Na^+^ (red), PAF-Mg^2+^ (blue), PAF-Fe^3+^ (magenta). (test conditions: the pH was 3, the adsorption temperature was 25 °C, the initial concentration of PFOS was 100 ng L^−1^ and the initial concentration of cations were 0.5 mM).


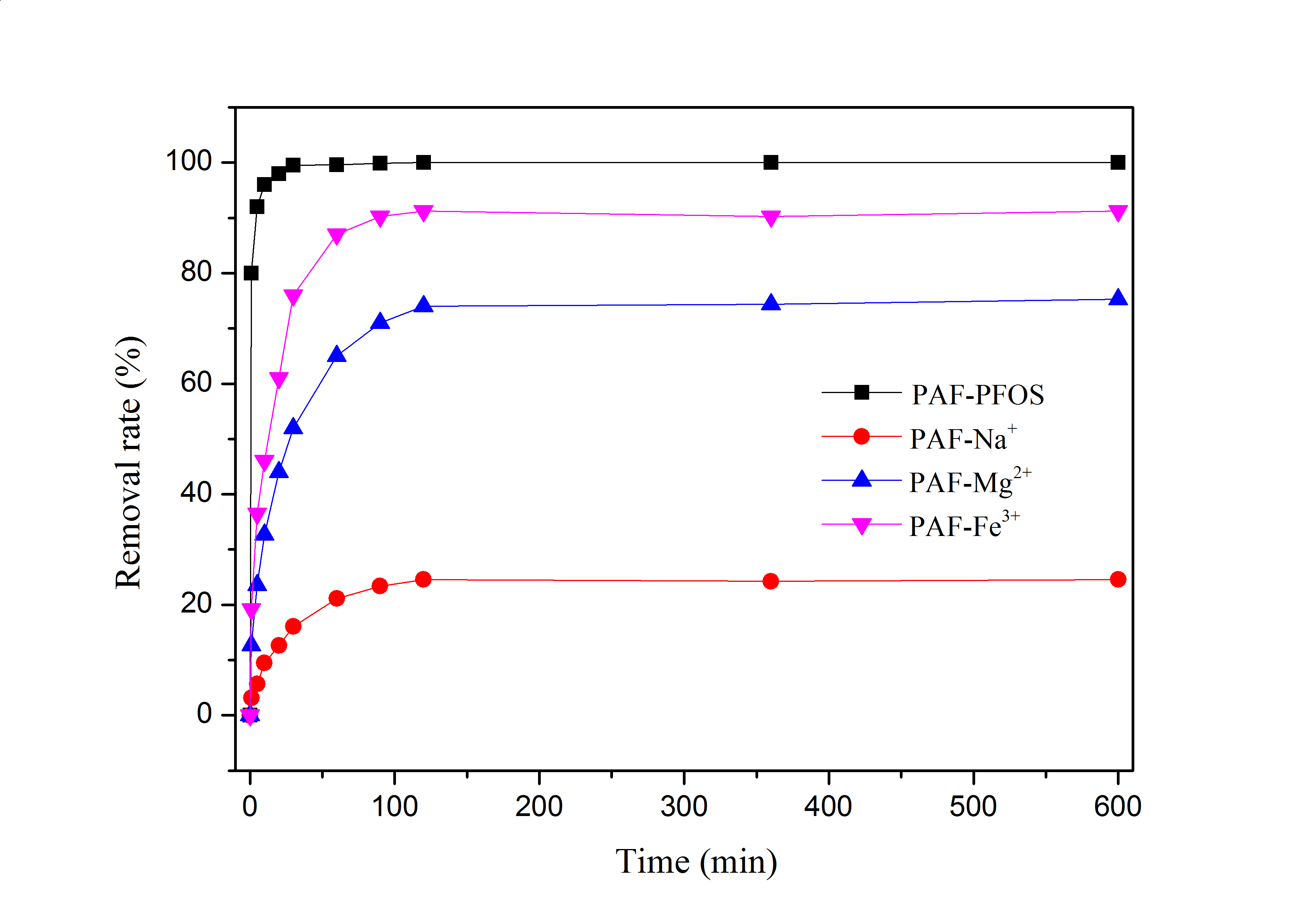


Figure S8 | Reaction kinetics of PAF-PFOS (black), PAF-Na^+^ (red), PAF-Mg^2+^ (blue), PAF-Fe^3+^ (magenta). (test conditions: the pH was 3, the adsorption temperature was 25 °C, the initial concentration of real PFOS was 128 ng L^−1^ , the initial concentration of Na^+^ was 0.23 mM, the initial concentration of Mg^2+^ was 0.74 mM, the initial Fe^3+^ concentration of Fe^3+^ was 0.98 mM).
